# Fibroblast growth factor homologous factor 1 stimulates Leydig cell regeneration from stem cells in male rats

**DOI:** 10.1111/jcmm.14461

**Published:** 2019-06-20

**Authors:** Jiaying Mo, Xiuxiu Chen, Chaobo Ni, Keyang Wu, Xiaoheng Li, Qiqi Zhu, Leika Ma, Yong Chen, Song Zhang, Yiyan Wang, Qingquan Lian, Ren‐Shan Ge

**Affiliations:** ^1^ Department of Obstetrics and Gynecology The Second Affiliated Hospital and Yuying Children's Hospital of Wenzhou Medical University Wenzhou China; ^2^ Department of Anesthesiology The Second Affiliated Hospital and Yuying Children's Hospital of Wenzhou Medical University Wenzhou China

**Keywords:** differentiation, FHF1, Leydig cells, proliferation, regeneration, testosterone

## Abstract

Fibroblast growth factor homologous factor 1 (FHF1) is an intracellular protein that does not bind to cell surface fibroblast growth factor receptor. Here, we report that FHF1 is abundantly present in Leydig cells with up‐regulation during its development. Adult male Sprague Dawley rats were intraperitoneally injected with 75 mg/kg ethane dimethane sulphonate (EDS) to ablate Leydig cells to initiate their regeneration. Then, rats daily received intratesticular injection of FHF1 (0, 10 and 100 ng/testis) from post‐EDS day 14 for 14 days. FHF1 increased serum testosterone levels without affecting the levels of luteinizing hormone and follicle‐stimulating hormone. FHF1 increased the cell number staining with HSD11B1, a biomarker for Leydig cells at the advanced stage, without affecting the cell number staining with CYP11A1, a biomarker for all Leydig cells. FHF1 did not affect PCNA‐labelling index in Leydig cells. FHF1 increased Leydig cell mRNA (*Lhcgr*, *Scarb1*, *Star*, *Cyp11a1*, *Hsd3b1*, *Cyp17a1*, *Hsd17b3, Insl3*, *Nr5a1* and *Hsd11b1*) and their protein levels in vivo. FHF1 increased preadipocyte biomarker *Dlk1* mRNA level and decreased fully differentiated adipocyte biomarker (*Fabp4* and *Lpl*) mRNA and their protein levels. In conclusion, FHF1 promotes Leydig cell regeneration from stem cells while inhibiting the differentiation of preadipocyte/stem cells into adipocytes in EDS‐treated testis.

## 1 INTRODUCTION

Adult Leydig cells (ALCs) are the testicular testosterone (T)‐producing cells, which play a vital role in the male reproductive system. The postnatal development of ALCs is required for the initiation and maintenance of spermatogenesis as well as for the promotion of the male secondary sexual characteristics.^1^ ALCs are derived from stem Leydig cells (SLCs), which exist near peritubular myoid cells.^1^ SLCs are spindle‐shaped cells and they differentiate into ALCs when ALCs wear off.^1^ When ALCs are dramatically damaged such as in the condition of ethane dimethane sulphonate (EDS)‐treated induction of ALC depletion, SLCs rapidly amplify their number via mitosis and then differentiate into ALCs, a process called Leydig cell (LC) regeneration.^2^, ^3^, ^4^, ^5^ When SLCs enter the LC lineage, they acquire luteinizing hormone (LH) receptor (LHCGR) for receiving LH trophic stimulation and express cholesterol transport proteins, high‐density lipoprotein receptor (SCARB1) and steroidogenic acute regulatory protein (STAR), as well as steroidogenic enzymes, including cytochrome P450 cholesterol side chain cleavage enzyme (CYP11A1), 3β‐hydroxysteroid dehydrogenase isoform 1 (HSD3B1), cytochrome P450 17α‐hydroxylase/17,20‐lyase (CYP17A1) and 17β‐hydroxysteroid dehydrogenase isoform 3 (HSD17B3).^1^ ALCs also secrete insulin‐like 3 to regulate spermatogenesis.^6^ The LC regeneration is very similar to the LC developmental process during puberty.^7^ SLCs begin to commit into progenitor Leydig cells (PLCs) on post‐EDS day 14 and then they further differentiate into immature Leydig cells (ILCs) on post‐EDS day 28, when the biomarker 11β‐hydroxysteroid dehydrogenase isoform 1 (HSD11B1) begins to be expressed in these advanced cells.^7^

The proliferation and differentiation of SLCs in the LC lineage is controlled by a set of growth factors and hormones.^1^ Although several critical growth factors such as platelet‐derived growth factor AA,^8^ dessert hedgehog,^9^ and kit ligand,^10^ have been reported, the regulatory growth factors are largely unknown.

By re‐analyzing the transcriptome of LC lineage cells,^11^ we identify fibroblast growth factor homologous factor 1 (FHF1), which is significantly up‐regulated from SLCs/PLCs into ILCs and further into ALCs, indicating that this peptide might exert autocrine effects on LC development. FHFs form a subfamily of proteins, which have sequences and structures similar to fibroblast growth factors (FGFs).^12^ FHFs are sometimes named according to FGF nomenclature (FHF1 = FGF12, FHF2 = FGF13, FHF3 = FGF11, FHF4 = FGF14). However, FHFs and FGFs have unrelated functions. FGFs bind to the cell surface receptor with tyrosine kinase activity.^13^ However, FHFs are expressed as intracellular peptides that bind to some intracellular proteins^14^ or cytoplasmic tails of some ion channels.^15^, ^16^ FHF members play a critical role in developmental cell processes and neuron excitability.^15^, ^17^ FHF1 is one of the FHF members that are widely expressed in many tissues such as cartilaginous skeleton, neuron, heart and testis, suggesting a role in the development of these tissues.^14^, ^18^ Here, we report that FHF1 stimulates LC development in rats.

## 2 RESULTS

### 2.1 FHF1 increases serum T levels in vivo

**Figure 1:**
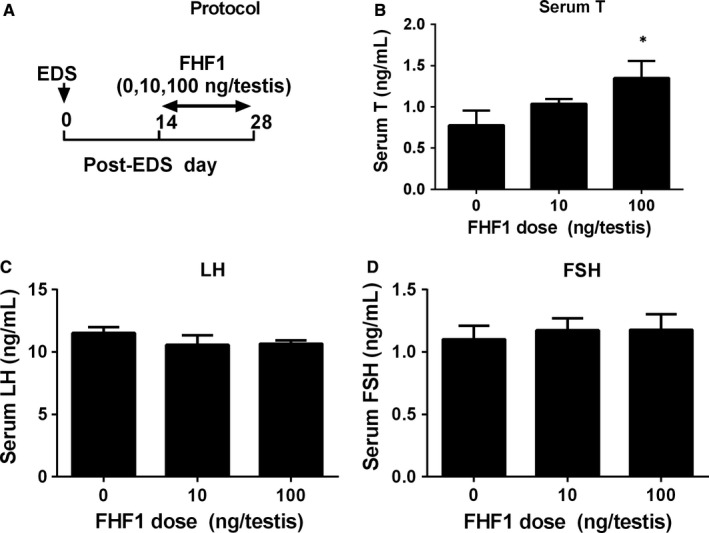
FHF1 experimental protocol and serum testosterone (T), LH and FSH levels after in vivo FHF1 treatment. A, Experimental protocol; B‐D, Serum T, LH, and FSH levels. Mean ± SEM, n = 8. Asterisks (*) designate significant difference from the control (FHF1, 0 ng/testis) at *P* < 0.05 respectively

After re‐analyzing the transcriptome of SLCs, PLCs, ILCs and ALCs,^11^ we found that the main expression levels (arbitrary unit) of *Fhf1* (*Fgf12*) in SLCs, PLCs, ILCs and ALCs were 46.13, 40.62, 130.12 and 213.18 respectively, suggesting that FHF1 is up‐regulated during LC development. We used an EDS‐treated LC regeneration model to study the function of FHF1 in vivo. Seven days after EDS, all LCs in the testis were eliminated, whereas SLCs were present.^19^ On post‐EDS day 14, PLCs reappeared, which were formed from the commitment of SLCs^4^ and on post‐EDS day 28, the PLCs differentiated into ILCs.^20^, ^21^ We intratesticularly injected FHF1 (0, 10 or 100 ng/testis/day) starting on post‐EDS day 14 up to day 28 (Figure 1A). After the treatment, FHF1 did not affect bodyweights and testis weights when compared to the control (Table S1). FHF1 dose‐dependently increased serum T levels with significance recorded at 100 ng/testis on post‐EDS day 28 (Figure 1B). However, it did not alter serum LH (Figure 1C) and FSH (Figure 1D) levels. These data suggest that FHF1 promotes LC regeneration primarily via direct action within the testis.

### 2.2 FHF1 increases HSD11B1‐positive LC number in vivo

**Figure 2:**
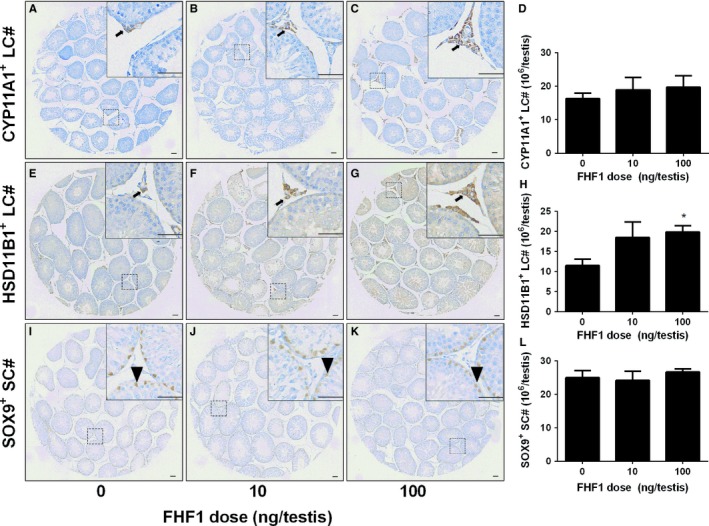
Leydig cell (LC) and Sertoli cell (SC) numbers in the testes after in vivo FHF1 treatment Immunohistochemical staining of CYP11A1 (Panels A‐C), HSD11B1 (Panels E‐G) and SOX9 (Panels I‐K) of the testes from the rats treated with 0, 10 and 100 ng/testis FHF1 on post‐EDS day 28. Panels A, E and I: the control; Panels B, F and J: 10 ng/testis FHF1; Panels C, G and K: 100 ng/testis FHF1; Panel D, H and L: quantitative data. Black arrow indicates CYP11A1‐ and HSD11B1‐positive LCs and black arrowhead indicates SOX9‐positive SCs. Bar = 50 mm. Mean ± SEM, n = 8, * *P* < 0.05 when compared to the control

Elevation of serum T levels could be contributed by the increase of LC number at the advanced stage. We used two biomarkers to label LCs: CYP11A1 (representing all LCs) and HSD11B1 (representing LCs at the advanced stage).^7^, ^20^ As shown in Figure 2, FHF1 did not change the number of CYP11A1‐positive LCs. However, FHF1 significantly increased the number of HSD11B1‐positive LCs at 100 ng/testis. This indicates that more LCs reach the advanced stage in the LC lineage after FHF1 treatment. We also measured SOX9‐positive cells and we found that FHF1 did not alter SOX9‐positive Sertoli cell number (Figure 2L).

### 2.3 FHF1 does not increase the PCNA labelling index in LCs in vivo

We used PCNA to label the proliferating cells and CYP11A1 to stain all LCs. As shown in Figure S1, we did not find that FHF1 increased the ratio of PCNA labelling in CYP11A1‐positive LCs. These results indicate that the increased number of HSD11B1‐positive cells is not contributed by LC proliferation but possibly by its differentiation.

We asked whether the increased number of HSD11B1‐positive LCs came from the proliferation of PLCs. PLCs have a higher capacity of cell division.^22^ We isolated PLCs and performed cell cycle analysis after in vitro FHF1 treatment. FHF1 did not affect the percentage of cells entering the S and G2 phases (Figure S2). The result indicates that FHF1 does not alter PLC mitosis.

### 2.4 FHF1 promotes LC differentiation in vivo

**Figure 3:**
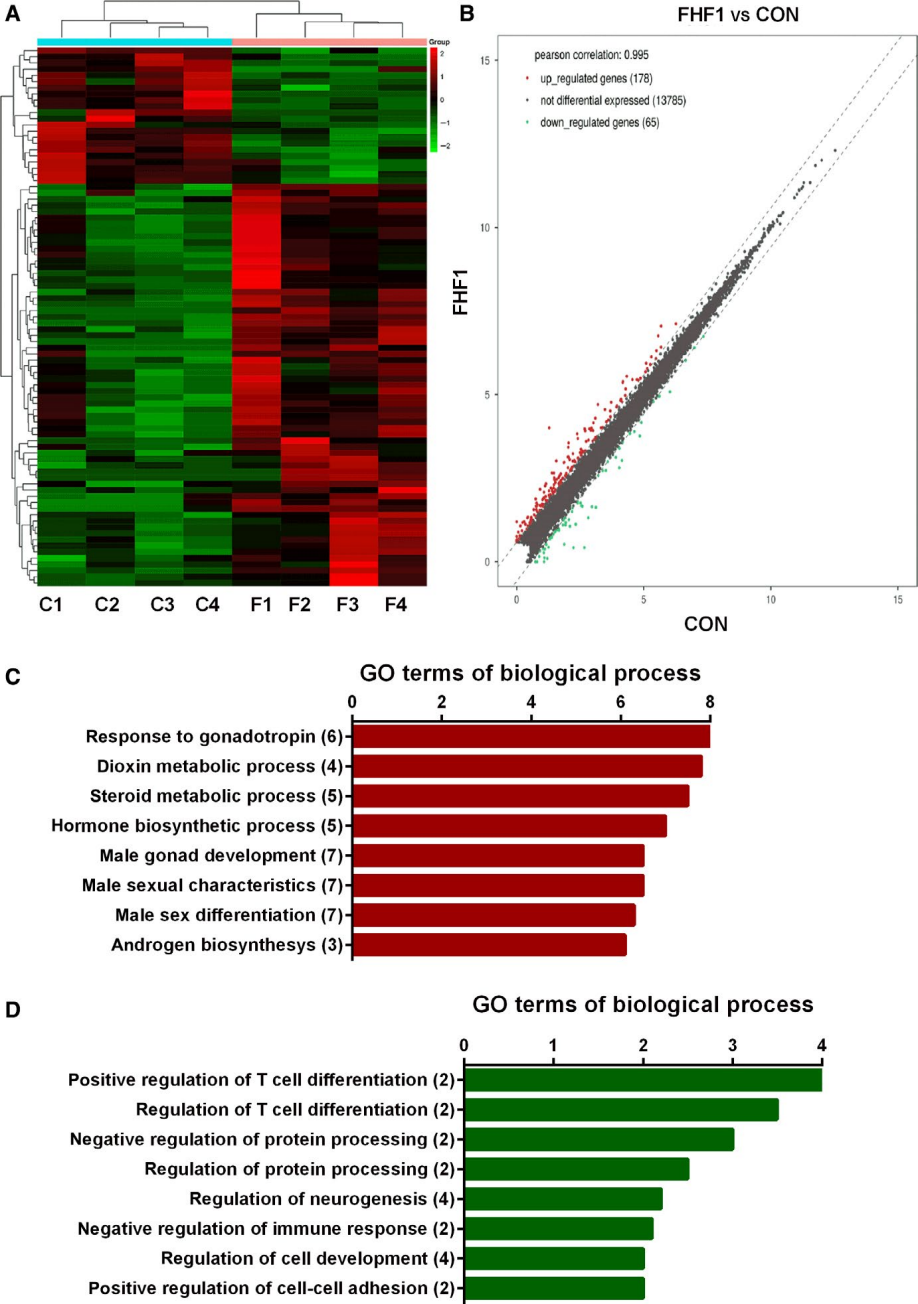
RNA‐seq analysis of FHF1‐treated testis. A, Heatmap of mRNAs between FHF1 (F, F1‐4) and control (C, C1‐4) samples; Red colour = up‐regulated genes, Green colour = down‐regulated genes; B, Scatter analysis of mRNAs between FHF1 and control (CON) samples; C, up‐regulated GO; D, down‐regulated GO. Mean ± SEM, n = 4

We performed RNA sequencing analysis to explore the effects of FHF1 (100 ng/testis) on LC gene expression. We sequenced 14,028 transcripts in the testis of two groups (0 and 100 ng/testis FHF1). Among these transcripts, 197 transcripts were significantly up‐regulated (*P* < 0.05) and 99 transcripts were significantly down‐regulated (*P* < 0.05) in the FHF1 group when compared to the control (Figure 3A,B). GO analysis showed that most up‐regulated genes are related to the categories including response to gonadotropin, dioxin metabolic process and steroid metabolic process (Figure 3C) and most down‐regulated genes are related to the categories including positive regulation of T cell differentiation, negative regulation of protein processing and regulation of cell development (Figure 3D).

**Figure 4:**
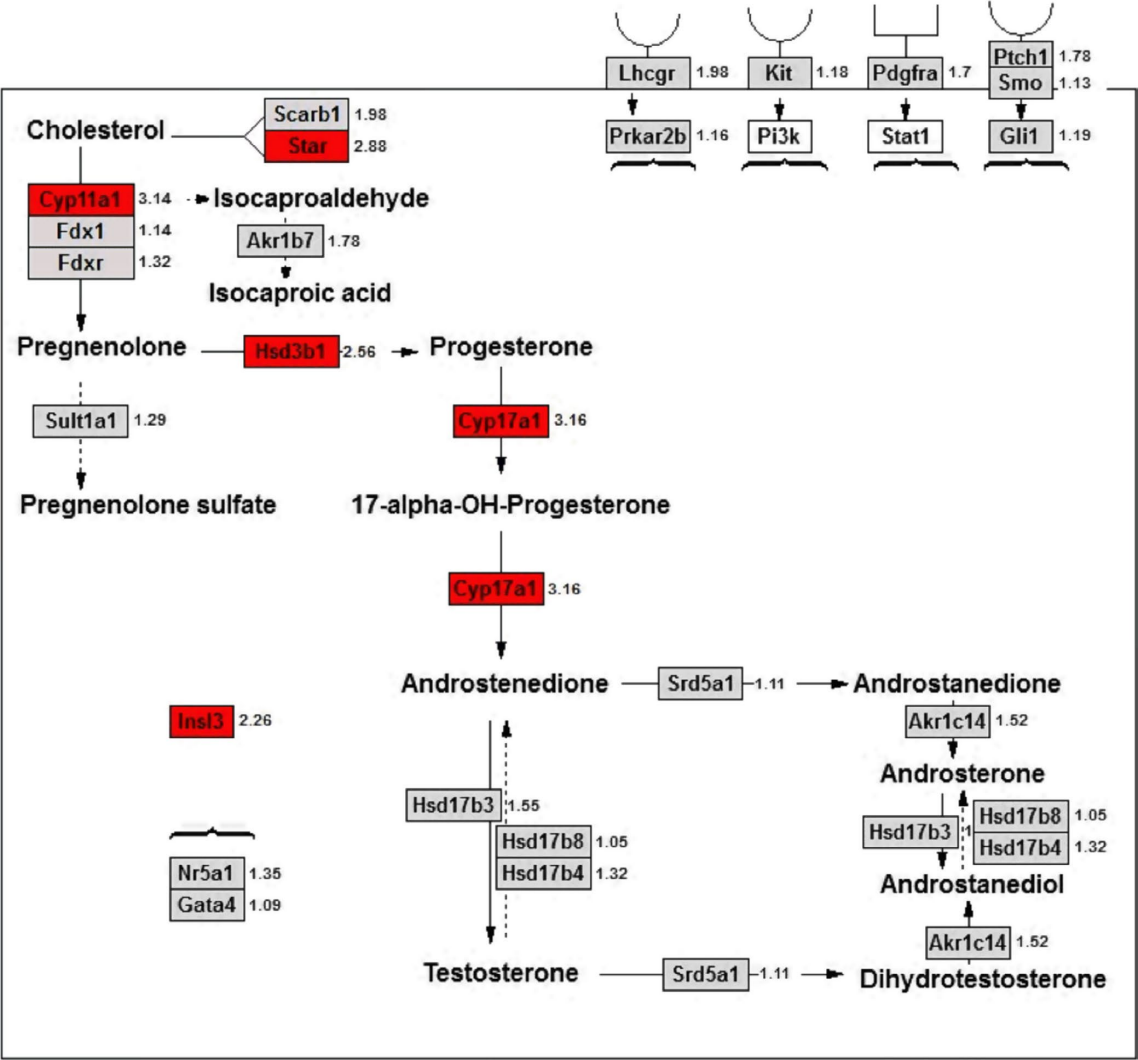
Gene pathway of Leydig cell (LC) steroidogenesis in FHF1‐treated testis. Red colour = up‐regulated genes at ≥2‐fold, green colour = down‐regulated genes at ≤2‐folds; grey colour = unchanged genes, white colour = unmapped genes; the digital number is the ratio of control over FHF1. Mean ± SEM, n = 4

**Figure 5:**
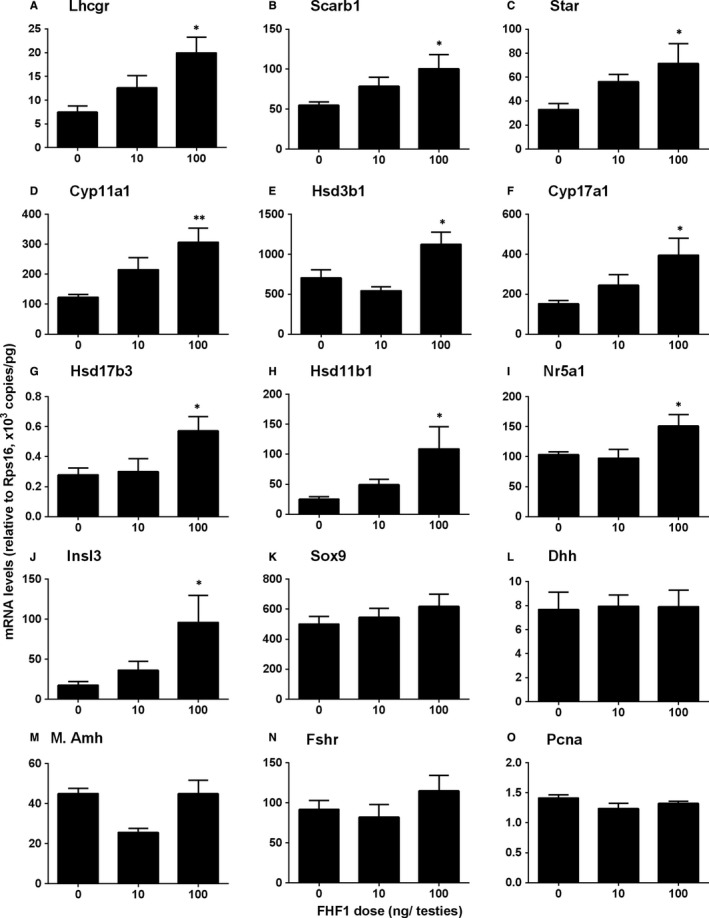
QPCR measurement of mRNA levels in the testes after in vivo FHF1 treatment Leydig cell genes: (A) *Lhcgr*, (B) *Scarb1*, (C) *Star*, (D) *Cyp11a1*, (E) *Hsd3b1*, (F) *Cyp17a1*, (G) *Hsd17b3*, (H) *Hsd11b1,* (I) *Nr5a1*, and (J) *Insl3*. Sertoli cell genes: (K) *Sox9*, (L) *Dhh*, (M) *Amh*, and (N) *Fshr*. Proliferaring gene: (O) *Pcna*. Mean ± SEM, n = 8. Asterisks (*, **) designate significant differences from the control (FHF1, 0 ng/testis) at *P* < 0.05 and 0.01 respectively

In the LC steroidogenic pathway, gene expression of LC genes (*Star*, *Cyp11a1*, *Hsd3b1*, *Cyp17a1* and *Insl3*) was up‐regulated by ≥2‐fold (Figure 4). We further verified the LC gene expression and compared it to the Sertoli cell genes using qPCR. As shown in Figure 5, all these LC genes were up‐regulated whereas Sertoli cell genes (*Sox9*, *Fshr*, *Amh* and *Dhh*) were not altered.

**Figure 6:**
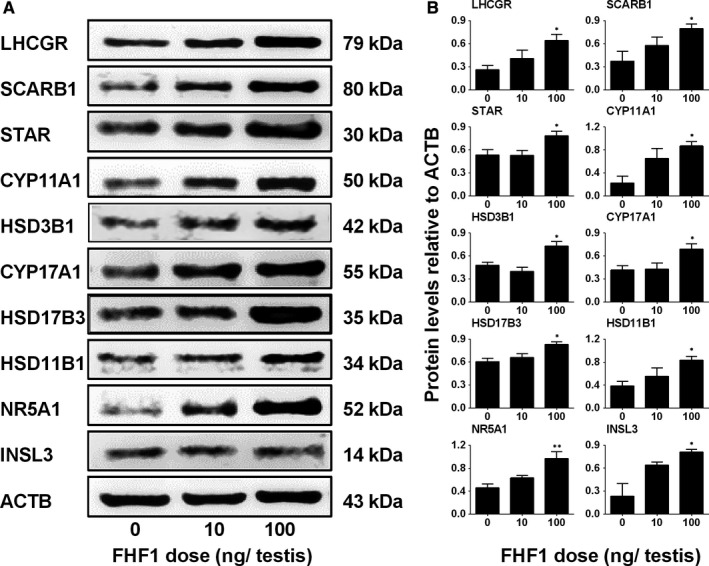
Protein levels of Leydig cells after in vivo FHF1 treatment Leydig cell (LC) proteins: A, Western blot band of LC proteins; B, Quantification of protein levels. Mean ± SEM, n = 8. Asterisks (*, **) designate significant differences from the control (FHF1, 0 ng/testis) at *P* < 0.05 and 0.01 respectively

**Figure 7:**
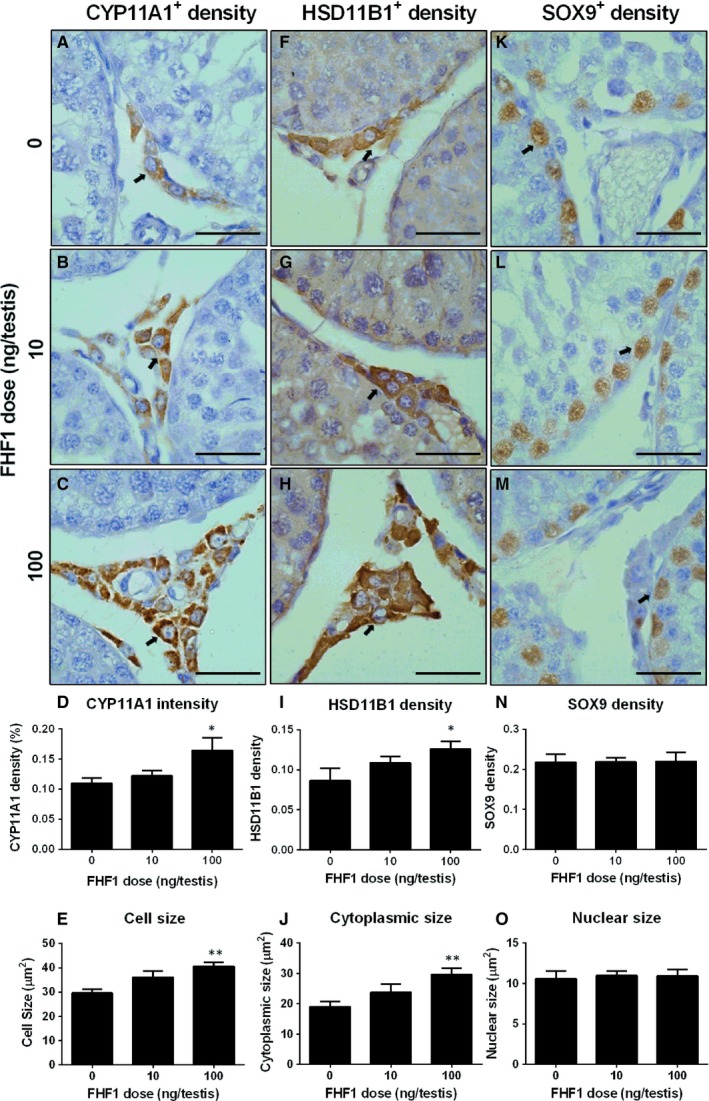
Semi‐quantitative measurement of CYP11A1, HSD11B1 and SOX9 levels and Leydig cell (LC) metrics in the testes after in vivo FHF1 treatment Immunohistochemical staining of CYP11A1 (Panels A‐C), HSD11B1 (Panels F‐H) and SOX9 (Panels K‐M) of the testes from the rats treated with 0, 10 and 100 ng/testis FHF1 on post‐EDS day 28. Panels A, F and K: the control (0 ng/testis FHF1); Panels B, G and L: 10 ng/testis FHF1; Panels C, H, and M: 100 ng/testis FHF1; Panels D, I, and N: quantitative data of protein density; Panels E, J and O: LC size, cytoplasmic size and nuclear size. Black arrow indicates CYP11A1, HSD11B1‐positive LCs and SOX9‐positive Sertoli cells. Mean ± SEM, n = 8, Asterisks (*, **) designate significant differences from the control (FHF1, 0 ng/testis) at *P* < 0.05 and 0.01 respectively

We measured the levels of these LC gene products using Western blot. We found that these proteins had similar changes in their respective mRNA levels (Figure 6). In addition, we also used the semi‐quantitative measurement of CYP11A1, HSD11B1, and SOX9 densities in the testis and we found that their densities were similar to the Western blotting data (Figure 7).

When SLCs and PLCs differentiate into ILCs, they increase cell size and cytoplasmic size.^23^ We used CYP11A1 to stain LCs to measure the LC metrics. As shown in Figure 7E,J,O, FHF1 significantly increased LC size and cytoplasmic size without affecting the nuclear size in the 100 ng/kg group, indicating that FHF1 stimulates the growth of LCs. These data suggest that FHF1 promotes LC regeneration.

### 2.5 FHF1 prevents the transition of stem cells into adipocyte in vivo

**Figure 8:**
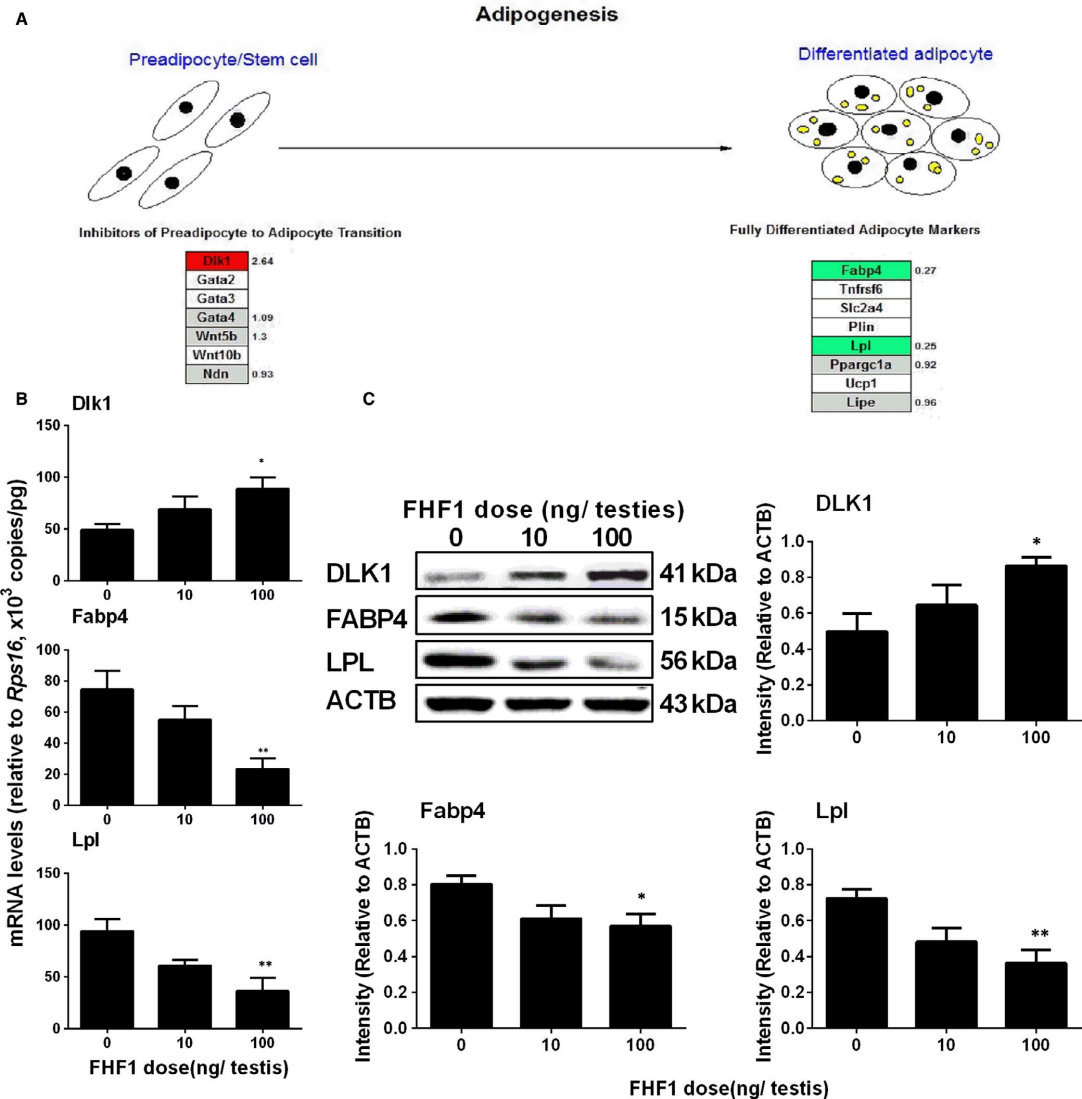
Gene expression in preadipocyte differentiation after FHF1 treatment. A, Signalling pathway analysis: the expression of inhibitor of preadipocyte to adipocytes' transition (*Dlk1*) was significantly up‐regulated whereas the markers of fully differentiated adipocytes (*Fabp4* and *Lpl*) were significantly down‐regulated. Red colour = up‐regulated genes at ≥2‐fold, green colour = down‐regulated genes at ≥2‐fold; grey colour = unchanged genes; white colour = unmapped genes. B, The expression of genes *(Dlk1, Fabp4*, and *Lpl*) was analysed using qPCR FHF1‐treated testes. C, Protein levels of DLK1, FABP4 and LPL were measured using Western blot. Mean ± SEM, n = 8. Asterisks (*, **) designate significant differences from the control (FHF1, 0 ng/testis) at *P* < 0.05 and 0.01 respectively

Our previous study demonstrated that SLCs are multipotent stem cells and can differentiate into adipocytes.^24^ We performed a transcriptome analysis to check whether FHF1 prevents the differentiation of stem cells into the adipocyte lineage. We found that the inhibitor of preadipocyte to adipocyte transition, *Dlk1*, was significantly up‐regulated, whereas the markers of fully differentiated adipocytes (*Fabp4* and *Lpl*) were significantly down‐regulated (Figure 8A). We used qPCR to confirm the mRNA levels of these genes (*Dlk1, Fabp4* and *Lpl*) and Western blotting to measure the protein levels of their products. The results showed that *Dlk1* mRNA level was up‐regulated and *Fabp4* and *Lpl* mRNA levels were significantly down‐regulated (Figure 8B). The protein levels were in parallel with their respective mRNA levels (Figure 8C). These results indicate that FHF1 promotes the differentiation of SLCs into the LC lineage by inhibiting the differentiation of preadipocyte/stem cells into adipocytes.

### 2.6 FHF1 regulates the signalling pathways

**Figure 9:**
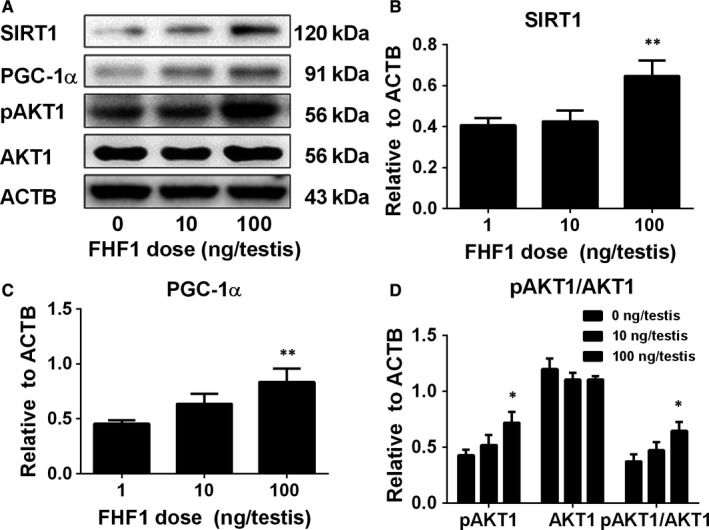
Signalling protein levels after in vivo FHF1 treatment Signalling proteins: A, Western blot bands; B, Quantification of protein levels. Mean ± SEM, n = 5. Asterisks (*, **) designate significant differences from the control (FHF1, 0 ng/testis) at *P* < 0.05 and 0.01 respectively

**Figure 10:**
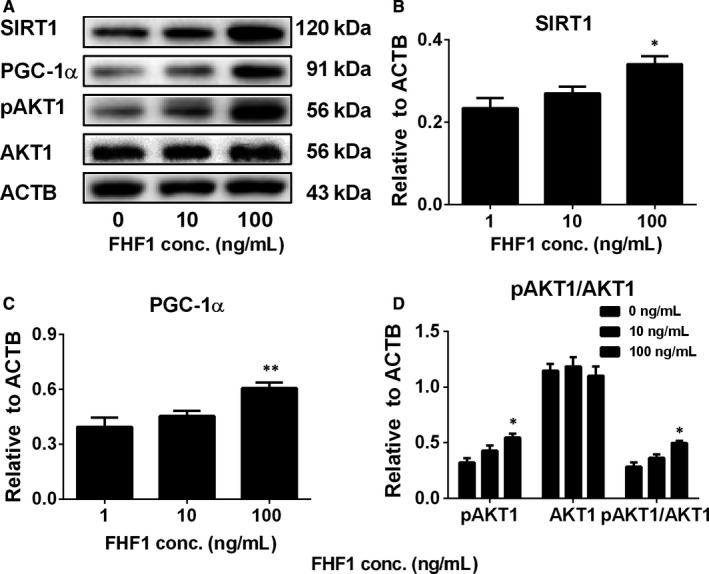
Signalling protein levels after in vitro FHF1 treatment to PLCs Signalling proteins: A, Western blot bands; B, Quantification of protein levels. Mean ± SEM, n = 5. Asterisks (*, **) designate significant differences from the control (FHF1, 0 ng/mL) at *P* < 0.05 and 0.01 respectively

We measured the levels of total proteins (SIRT1, PGC‐1α and AKT1) and the phosphorylated protein (pAKT1) in the testis after FHF1 treatment. AKT1 level was not changed, whereas phosphorylated AKT1 (pAKT1) levels were significantly increased at 100 ng/testis FHF1 group (Figure 9). FHF1 significantly increased SIRT1 and PGC‐1α levels at 100 ng/testis (Figure 9). We further treated PLCs with 10 and 100 ng/mL FHF1 for 24 hours and found that FHF1 significantly increased pAKT1 and SIRT1 and PGC‐1α levels at 100 ng/mL (Figure 10). This indicates that phosphorylated AKT1 and SIRT1/PGC‐1α signalling pathways are involved in the FHF1‐mediated action in LC regeneration.

## 3 DISCUSSION

Although classic FGFs belong to a family of signalling proteins that bind to the cell surface FGF receptors and play diverse roles in cell growth, differentiation, morphogenesis and developmental processes,^25^, ^26^, ^27^, ^28^ FHFs are not exact FGF members and are intracellular non‐secretory proteins.^29^ FHFs lack a signal sequence and cannot be released from cells^30^ to activate FGF receptors.^31^ FHF1 binds to islet brain‐2 and voltage‐gated sodium channels and plays a critical role in the membrane targeting and ion channel function.^14^ Here, we report that FHF1 is developmentally up‐regulated in the LC lineage and promotes LC regeneration by inducing their differentiation.

Serum androgen levels depend on the steroidogenic capacity of LCs and the number of these cells. In the adult rat testis, about 25 million ALCs reside in each testis and they respond to the stimulation of LH to produce T.^32^ LC number is achieved by the mitosis of SLCs and PLCs and their subsequent differentiation.^1^ We used the LC regeneration model after EDS injection to examine FHF1 function and its mechanisms. EDS depletes all LCs in adult rat testis without affecting SLCs,^5^ thus initiating LC regeneration.^33^, ^34^, ^35^ CD90‐positive SLCs isolated from the surface of seminiferous tubules after EDS treatment can be induced to differentiate into LCs in vitro.^9^ This suggests that SLCs in the EDS‐treated model are CD90‐positive cells and are homogenous. We treated rats with 10 and 100 ng/testis FHF1 on post‐EDS day 14 when SLCs begin to commit into the LC lineage. We selected these doses of FHF1 based on previous publication for doses of FGF1^36^ and FGF16.^37^ Since the microarray showed that rat testis expressed FGF1, FHF1, and FGF16 with equivalent amounts, the doses of FHF1 might be comparable to those of FGF1 and FGF16. Apparently, FHF1 increased serum T level without affecting serum LH and FSH levels at 100 ng/mL (Figure 1). Previous studies demonstrated that several classic members of the FGF family, FGF2 and FGF16, were able to bind to FGF receptors, thus stimulating SLC and PLC mitosis and inhibiting LC steroidogenesis and regeneration.^9^, ^38^, ^39^ Unlike these classic FGFs, FHF1 instead did not affect LC proliferation, as shown by the unchanged number of CYP11A1‐positive LCs (Figure 2), unaltered PCNA‐labelling index in LCs (Figure S1) and unchanged cell cycle in PLCs (Figure S2). However, FHF1 increased HSD11B1‐positive LC number (Figure 2). This increase could be contributed by the differentiation of SLCs/PLCs into LCs at the advanced stage. Indeed, FHF1 increased the expression of *Lhcgr, Scarb1, Star, Cyp11a1, Hsd3b1, Cyp17a1, Hsd17b3, Nr5a1* and *Hsd11b1* and their proteins (Figure 5 and Figure 6).

Unlike the classic FGFs, FHF1 should enter the cells via endocytosis. Although the endocytosis efficiency of FHF1 is unknown, the previous study showed that FHF1 showed the dose‐dependent increase of endocytosis to intestinal epithelial cells when 10, 100 and 1000 ng/mL FHF1 were added and after 24‐48 hours, the endocytosis reached a maximum and about 96% of cells showed FHF1 endocytosis.^40^

The previous study demonstrated that SLCs are multipotent stem cells and are able to differentiate into either the LC lineage or the adipocyte lineage. 24 Using RNA sequencing and qPCR as well as Western blotting, we found that biomarkers of mature adipocytes (*Fabp4* and *Lpl*) were significantly down‐regulated whereas *Dlk1* (the biomarker of preadipocytes) and its protein levels were remarkably up‐regulated (Figure 8). DLK1, encoded by *Dlk1*, is a non‐canonical NOTCH1 ligand that inhibits NOTCH1 signalling in a dose‐dependent manner and modulates the adipogenesis process of 3T3‐L1 preadipocytes.^41^ FHF1 inhibits the differentiation of preadipocyte/stem cells into adipocytes, whereas it promotes the differentiation of SLCs into the LC lineage.

We further explored the possible pathways that FHF1 might be involved in. Previous studies demonstrated that the AKT1 signalling pathway is involved in LC development.^42^ AKT1 regulates numerous cellular processes, including cell differentiation, proliferation and apoptosis.^43^ AKT1 knockout in mice causes morphological abnormalities of mouse testis.^44^ Another growth factor, insulin‐like growth factor 1 (IGF‐1) also exerts to stimulate LC differentiation via increasing the phosphorylation of AKT1.^45^ Indeed, IGF‐1 null mice exhibited significant down‐regulation of LC genes, such as *Star*, *Cyp11a1*, *Hsd3b1* and *Cyp17a1* and the reduced T synthesis.^46^, ^47^

The SIRT1/PGC‐1α signalling may also be involved in FHF1‐mediated action. SIRT1 is an NAD‐dependent class III histone deacetylase and plays significant roles in many biological activities, including development, gene modification and metabolism via deactivation.^48^ SIRT1 targets PGC‐1α, a transcriptional co‐activator, which promotes β‐oxidation of fatty acids for the generation of energy.^49^ SIRT1 interacts with PGC‐1α and deacetylates it to increase PGC‐1α activity, thus promoting the biogenesis of mitochondria, which not only provide energy but also provide the space for steroidogenic enzyme CYP11A1.^50^ SIRT1 and PGC‐1α are present in the LCs of rodents, indicating that they regulate the mitochondrial biogenesis and steroidogenesis.^49^ Indeed, the present study demonstrated that FHF1 increased pAKT1, SIRT1 and PGC‐1α levels both in vivo (Figure 9) and in vitro (Figure 10), suggesting that FHF1 regulates Leydig cell development by increasing the levels of these proteins.

NR5A1 may also be involved in FHF1‐mediated action. FGF1 in vivo significantly increased NR5A1 mRNA and protein levels. NR5A1 is a ligand‐free nuclear receptor and is a critical transcription factor for promoting LC development. NR5A1 can bind to the promoters of many LC‐specific genes such as *Star*, *Cyp11a1*, *Cyp17a1* and *Hsd3b1*.^51^, ^52^, ^53^, ^54^ Null mutation of NR5A1 caused LC agenesis.^55^ Forced expression of NR5A1 can even convert stem cells or fibroblasts into steroidogenic LC‐like cells by transcriptionally promoting the expression of LHCGR and other steroidogenic enzymes (CYP11A1, HSD11B1, CYP17A1 and HSD17B3).^56^

In conclusion, unlike classic FGFs, which inhibit LC differentiation and stimulate LC proliferation, FHF1 promotes LC differentiation without affecting their proliferation. FHF1 is possibly involved in several signalling pathways, including SIRT1/PGC‐1α and NR5A1 signalling.

## 4 MATERIALS AND METHODS

### 4.1 Chemicals and kits

FHF1 was purchased from GenScript (Catalogue number Z03129‐50; Piscataway, NJ). Immulite2000 Total T kit was purchased from Sinopharm (Hangzhou, Zhejiang, China). EDS was purchased from Pterosaur Biotech Co. (Hangzhou, China).

### 4.2 Animals and treatments

Twenty‐four adult (60‐day‐old) male Sprague Dawley rats were purchased from Shanghai Laboratory Animal Center (Shanghai, China). After one‐week adjustment, each rat received a single intraperitoneal injection of EDS (75 mg/kg bodyweight/once), which was dissolved in a mixture of DMSO: H_2_O (1:3, v/v), to eliminate LCs from rat testis. LC‐depleted rats were randomly divided into three groups with eight rats per group. FHF1 was dissolved in normal saline for injection. Each rat daily received intratesticular injection (20 μL) of 0, 10 or 100 ng/testis FHF1 for 14 days, starting on post‐EDS day 14. This time‐course of administration regimen was adopted because PLCs begin to emerge from SLCs on post‐EDS day 14.^7^ On post‐EDS day 28, rats were killed and the blood samples were collected. The serum sample was stored at −20°C to investigate T, LH and FSH. One testis per rat was frozen in −80°C for mRNA and protein expression study. The contralateral testis was punched and fixed in Bouin's solution for immunohistochemical and immunofluorescent staining processes. All animal procedures were performed in accordance with the protocol approved by the Animal Care and Use Committee of Wenzhou Medical University.

### 4.3 Serum T measurement

Immulite2000 Total T kit was employed to measure the serum T concentrations. The lower detection limit of serum T concentrations was 0.2 ng/mL.

### 4.4 ELISA for serum LH and FSH levels

According to the manufacturer's instructions (Chemicon CA), each ELISA kit was used to detect the serum levels of LH and FSH. Briefly, sample and assay diluent was mixed, washed and incubated with peroxidase‐conjugated IgG anti‐LH or anti‐FSH. Then, the conjugated complex was washed and substrate was added for the reaction. The levels of LH and FSH were measured at 550 nm using a microplate reader with a correction wavelength at 450 nm.

### 4.5 RNA sequencing

Sequencing analysis and base calling were conducted using Solexa pipeline v1.8 (Off‐Line Base Caller software, v1.8, Illumina, Foster City, CA). Sequence quality was examined using the FastQC software.^57^ The trimmed reads (trimmed 5′, 3′‐adaptor bases) were aligned to a reference genome using Hisat2 software.^58^ The transcript abundance for each sample was estimated with StringTie^59^ and the FPKM^60^ value for gene and transcript levels were calculated with R package Ballgown.^61^ The differentially expressed genes and transcripts were filtered using R package Ballgown.^61^ The novel genes and transcripts were predicted from assembled results by comparing to the reference annotation using StringTie and Ballgown and the coding potential of those sequences was then assessed using CPAT.^60^ Principle Component Analysis and correlation analysis were performed according to gene expression level. Hierarchical Clustering, Gene Ontology, Pathway analysis, scatter plots and volcano plots were performed with the differentially expressed genes in R, Python or shell environment for statistical computing and graphics.

### 4.6 Biological pathway analysis

Biological pathway analysis was performed as previously described.^62^ GenMAPP2.1 (San Francisco, CA) was used to map the signal pathways of potential pathways. We illustrated the biological pathways containing differentially expressed genes by importing our statistical results into the program. The results of differential gene expression profiles were confirmed using qPCR.

### 4.7 qPCR

Total RNAs were isolated from testes using Trizol. The concentrations of total RNAs were measured using NanoDrop 2000 (Thermo Scientific, Shanghai, China). The first‐strand cDNA was synthesized and used as the template for qPCR as previously described.^63^ SYBR Green qPCR kit (Roche, Basel, Switzerland) was used to analyse the LC (*Lhcgr, Scarb1, Star, Cyp11a1, Hsd3b1, Cyp17a1, Hsd17b3, Hsd11b1* and* Nr5a1*) and the Sertoli cell (*Fshr, Sox9, Amh* and *Dhh*) mRNA levels. The PCR reaction mixture consisted of SYBR Green mix, primers, cDNAs and RNase‐free water. The procedure for qPCR was set as following: 95°C for 5 minutes, followed by 40 cycles of 95°C for 10 seconds and 60°C for 30 seconds. Ribosomal protein S16 (*Rps16*) was used as the internal control, which was the house‐keeping gene. The mRNA level of each gene was read as the Ct value and calculated using a standard curve method and was normalized to *Rps16* as previously described.^64^ The primers and gene names are listed in Table S2.

### 4.8 Western blot

Western blot was performed as previously described.^65^ Proteins were prepared from FHF1‐injected testes and PLCs. Tissues were homogenized and put into lysis buffer (Bocai Biotechnology, China) to obtain protein samples. The total protein concentrations of samples were measured using the BCA Protein Assay Kit (Takara, Japan). Total protein (30 μg) was loaded and electrophoresed on 10% polyacrylamide gels and then the separated proteins were transferred onto the nitrocellulose membranes. The membranes were blocked with 5% non‐fat milk in TBST buffer for 2 hours and incubated with primary antibodies against LHCGR, SCARB1, STAR, CYP11A1, HSD3B1, CYP17A1, HSD17B3, NR5A1, HSD11B1 and ACTB at 4°C overnight. After that, the membranes were washed and incubated with HRP‐conjugated anti‐rabbit (1:2000; Bioword, St. Louis Park, MN) for 2 hours at room temperature. The band was visualized by chemiluminescence using an ECL kit (Amersham, Arlington Heights, IL). The house‐keeping protein, ACTB, serves as a control. The density of target protein calculated by using J‐Software was normalized to ACTB. All the antibodies used were listed in Table S3.

### 4.9 Immunohistochemical staining of the testis and enumeration of LCs

Immunohistochemical staining was performed to investigate the effects of FHF1 on LC and Sertoli cell number. One testis from each rat was used for immunohistochemical staining (Vector Laboratories, Inc, Burlingame, CA) according to the manufacturer's instructions. Eight testes per group were randomly prepared as testis samples and then embedded in paraffin as a tissue array. Tissue array samples were dehydrated in ethanol and xylene and then embedded in paraffin. Six micrometre‐thick transverse sections were cut and mounted on glass slides. Antigen retrieval was conducted by microwave irradiation in 10 mM (pH 6.0) citrate buffer for 10 minutes. After that, endogenous peroxidase was blocked with 0.5% of H_2_O_2_ in methanol for 30 minutes. The sections were then incubated with CYP11A1 or HSD11B1 or SOX9 polyclonal antibody diluted 1:200 for 1 hour at room temperature. Diaminobenzidine was used for visualizing the antibody‐antigen complexes, positively labelling LCs by brown cytoplasmic staining or Sertoli cells by brown nuclear staining. Mayer haematoxylin was applied in the counterstaining. The sections were dehydrated in graded concentrations of alcohol and cover‐slipped with resin (Thermo Fisher Scientific, Waltham, UK). Non‐immune rabbit IgG was used in the incubation of negative control sections with working dilution the same as the primary antibody.

In order to enumerate CYP11A1‐ or HSD11B1‐positive LC numbers or SOX9‐positive Sertoli cell number, sampling of the testis was performed according to a fractionator technique as previously described.^66^ Briefly, each testis was cut in eight discs and two discs were randomly selected. Then, discs were cut into four pieces and one piece was randomly selected from a total of eight pieces. These pieces of testis were embedded in paraffin in a tissue array as above. Paraffin blocks were sectioned into 6‐μm‐thick sections. Ten sections were randomly sampled from each testis per rat. Sections were used for immunohistochemical staining. Images were taken using a digital camera, under a 10 × objective and total microscopic fields per section were counted. The histochemical staining was performed as above. The total number of LCs or Sertoli cells was calculated by multiplying the number of LCs or Sertoli cells counted in a known fraction of the testis by the inverse of the sampling probability.

### 4.10 Immunofluorescent staining of the testis

Immunofluorescent staining was performed to investigate the effects of FHF1 on the proliferation of LCs. Sections were incubated with the primary antibody of PCNA for 60 minutes and then washed and incubated with the CYP11A1 antibody for double staining. Fluorescent secondary antibody (Alexa‐conjugated anti‐rabbit or anti‐mouse IgG, 1:500) were used after the primary antibody. Sections were counterstained with mounting medium containing DAPI. Sections were visualized under a fluorescent microscope (Olympus, Tokyo, Japan). The CYP11A1 (green colour) was used to label LCs and PCNA (red colour) was used to label proliferating cell nucleus.

### 4.11 PLC isolation

PLCs were isolated as described previously.^67^ Eighteen 21‐day‐old Sprague Dawley rats were killed by asphyxiation with CO_2_. In brief, the removed testis was digested with collagenase and DNase (Sigma‐Aldrich, St. Louis, MO) for 15 minutes. Then, the cell suspension was filtered using a 100 μm nylon mesh to remove the tissue debris and the cells were separated under Percoll gradient. The cells with a density of 1.07‐1.088 g/mL were transferred into a new tube and washed. Purified cells were evaluated using histochemical staining for HSD3B1 activity, with 0.4 mM etiocholanolone as the steroid substrate.^68^ More than 95% of PLCs were stained, indicating that the purity of PLCs was high.

### 4.12 PLC culture and cell cycle assay

PLCs were seeded into the 6‐well culture plates after isolation with a cell density of 10^6^ cells/well. PLCs were treated with 0, 10 and 100 ng/mL FHF1 in 2.0 mL DMEM: F12 medium for 24 hours. Cells were harvested for analysis of the cell cycle using cytometry and the measurement of AKT1, pAKT1, SIRT and PGC‐1α levels using Western blotting. Then, the cells were harvested and fixed with 75% cold ethanol overnight at 4°C. The fixed cells were washed once with phosphate‐buffered saline (PBS) and stained darkly with propidium iodide (PI) for 30 minutes at room temperature. The stained cells were analysed using a flow cytometer (BD FACSAria, San Diego, CA).

### 4.13 Statistical analysis

Data are expressed as mean ± SEM. *P* < 0.05 was considered statistically significant. The differences among groups were evaluated by unpaired student *t* test when two groups were compared or by one‐way ANOVA followed by ad hoc Dunnett's multiple comparisons to compare with the control when three or more groups were compared. GraphPad version 6 software was used for statistical analysis (GraphPad Inc, CA).

## CONFLICT OF INTEREST

The authors declare that they have no conflict of interest to declare.
