# Supplementary material for: Fibroblast growth factor homologous factor 1 stimulates Leydig cell regeneration from stem cells in male rats
Source: J Cell Mol Med. 2019 Jun 20;23(8):5618–31. doi: 10.1111/jcmm.14461 (PMC6653537; doi:10.1111/jcmm.14461)
Supplement: Supplementary file 1 [file JCMM-23-5618-s001.doc]

**Supplementary Table S1. General parameters after treatment of FHF1**

| **Parameters** | | **FHF1 dose (ng/testis)** | | | |
| --- | --- | --- | --- | --- | --- |
|  |  | **0** | **10** | **100** |  |
| **Body weight (g)** | |  |  |  |  |
|  | Before | 273.6 ± 7.68 | 273.9 ± 8.55 | 269.8 ± 8.59 |  |
|  | After treatment | 323.8 ± 10.74 | 333.2 ± 9.61 | 325.8 ± 9.53 |  |
| **Testes weight (g)** | |  |  |  |  |
|  | After treatment | 1.38 ± 0.11 | 1.22 ± 0.14 | 1.14 ± 0.039 |  |

Mean ± SEM, n = 8. No significant difference was found in FHF1-treated groups from the control (0 ng/testis FHF1).
