# Supplementary material for: Fibroblast growth factor homologous factor 1 stimulates Leydig cell regeneration from stem cells in male rats
Source: J Cell Mol Med. 2019 Jun 20;23(8):5618–31. doi: 10.1111/jcmm.14461 (PMC6653537; doi:10.1111/jcmm.14461)
Supplement: Supplementary file 2 [file JCMM-23-5618-s002.doc]

**Supplementary Table S2**. Primer information

| **Primer**  **Symbol** | **Gene name** | **Primer direction** | **Sequences (5’to 3’)** | **PCR**  **(bp)** | **Accession** |
| --- | --- | --- | --- | --- | --- |
| Lhcgr | Luteinizing hormone receptor | Forward | CTGCGCTGTCCTGGCC | 103 | NM_012978 |
|  |  | Reverse | CGACCTCATTAAGTCCCCTGAA |  |  |
| Scarb1 | Scavenger receptor class B, member 1 | Forward | ATGGTACTGCCGGGCAGAT | 117 | NM_031541 |
|  |  | Reverse | CGAACACCCTTGATTCCTGGTA |  |  |
| Star | Steroidogenic acute regulatory protein | Forward | CCCAAATGTCAAGGAAATCA | 187 | NM_031558 |
|  |  | Reverse | AGGCATCTCCCCAAAGTG |  |  |
| Cyp11a1 | Cholesterol side chain cleavage enzyme | Forward | AAGTATCCGTGATGTGGG | 127 | NM_017286 |
|  |  | Reverse | TCATACAGTGTCGCCTTTTCT |  |  |
| Hsd3b1 | 3β-Hydroxysteroid dehydrogenase 1 | Forward | CCCTGCTCTACTGGCTTGC | 189 | NM_001007719 |
|  |  | Reverse | TCTGCTTGGCTTCCTCCC |  |  |
| Cyp17a1 | 17α-hydroxylase/ 17,20-lyase | Forward | TGGCTTTCCTGGTGCACAATC | 90 | NM_012753 |
|  |  | Reverse | TGAAAGTTGGTGTTCGGCTGAAG |  |  |
| Hsd17b3 | 17β-Hydroxysteroid dehydrogenase 3 | Forward | TGAAAGTTGGTGTTCGGCTGAAG | 202 | NM_054007 |
|  |  | Reverse | TGAAAGTTGGTGTTCGGCTGAAG |  |  |
| Hsd11b1 | [11β-Hydroxysteroid dehydrogenase 1](https://www.baidu.com/link?url=RASn5FVJOHQO5F8yLZuLK2GaE-txBvaDG-Aix0zS1TOK-H6BDM3SQ-dtmZqJCiib&wd=&eqid=adb6e42d00037867000000045854b8a0) | Forward | GAAGAAGCATGGAGGTCA | 290 | NM_017080 |
|  |  | Reverse | CTCAAGATTATCCCAGAGG |  |  |
| Nr5a1  Insl3 | Nuclear receptor 5 A1  Insulin-like 3 | Forward | CAGAGCTGCAAAATCGACAA | 186  102 | NM_053344  NM_053680 |
|  |  | Reverse  Forward  Reverse | CCCGAATCTGTGCTTTCTTC  GTGGCTGGAGCAACGACA  TGAAAGTTGGTGTTCGGCTGAAG |  |  |
| Fshr | Follicle stimulating hormone receptor | Forward  Reverse | CCACAAGCCAATACAAACTAACT  CAAAAGTCCAGCCCAATACC | 327 | NM_199237 |
| Sox9 | SRY box 9 | Forward | TGCTGAACGAGAGCGAGAAG | 160 | NM_080403 |
|  |  | Reverse | ATGTGAGTCTGTTCGGTGGC |  |  |
| Dhh  Amh | Desert hedgehog  Antimulerian hormone | Forward | AACCCCGACATAATCTTCA | 150  82 | NM_053367  NM_012902 |
|  |  | Reverse  Forward  Reverse | CTCGTCCCAACCTTCAGT  GCCCTAACCCTTCAACCA  GGGAATCAGAGCCAAACAGA |  |  |
| Rps16 | Ribosomal protein  S16 | Forward | AAGTCTTCGGACGCAAGAAA | 148 | [NM_001169146](https://www.ncbi.nlm.nih.gov/entrez/viewer.fcgi?db=nucleotide&id=310703681) |
|  |  | Reverse | TTGCCCAGAAGCAGAACAG |  |  |
| Pcna | Proliferating cell nuclear antigen | Forward  Reverse | AGGACGGGGTGAAGTTTTCT  CAGTGGAGTGGCTTTTGTGA | 173 | NM_022381.3 |
| Dlk1 | Delta like non-canonical Notch ligand 1 | Forward  Reverse | GGCCATCTGCTTCACCATCCTG  GCTCCTCGCCGCTGTTGTAC | 167 | NM_053744.1 |
| Fabp4 | Fatty acid binding protein 4 | Forward  Reverse | ACTTGGTCGTCATCCGGTCAGAG  CCACTTCTGCACATGTACCAGGAC | 152 | NM_053365.1 |
| Lpl | Lipoprotein lipase | Forward  Reverse | CTGGTGAAGTGCTCGCACGAG  CTGCTTCTCTTGGCTCTGACCTTG | 191 | NM_012598.2 |
