# Supplementary material for: Fibroblast growth factor homologous factor 1 stimulates Leydig cell regeneration from stem cells in male rats
Source: J Cell Mol Med. 2019 Jun 20;23(8):5618–31. doi: 10.1111/jcmm.14461 (PMC6653537; doi:10.1111/jcmm.14461)
Supplement: Supplementary file 3 [file JCMM-23-5618-s003.doc]

**Supplementary Table S3. Antibody information**

| **Antibody** | **Species** | **Vendor (City, State, catalogue)** | **Dilution** | |
| --- | --- | --- | --- | --- |
|  |  |  | **WB** | **HS** |
| ACTB | Rabbit | Cell Signaling Technology (Danvers, MA) | 1:1000 | ND |
| LHCGR | Rabbit | Multi Sciences (Hangzhou, China) | 1:1000 | ND |
| SCARB1 | Rabbit | Multi Sciences (Hangzhou, China) | 1:1000 | ND |
| STAR | Rabbit | Cell Signaling Technology (Danvers, MA) | 1:1000 | ND |
| CYP11A1 | Rabbit | Cell Signaling Technology (Danvers, MA) | 1:1000 | 1:500 |
| HSD3B1 | Mouse | Abcam (San Francisco, CA) | 1:1000 | ND |
| CYP17A1 | Rabbit | Abcam (San Francisco, CA) | 1:1000 | ND |
| HSD17B3 | Rabbit | Abcam (San Francisco, CA) | 1:1000 | ND |
| HSD11B1 | Rabbit | Abcam (San Francisco, CA) | 1:1000 | 1: 500 |
| NR5A1  INSL3 | Mouse  Rabbit | Cell Signaling Technology (Danvers, MA) Abcam (San Francisco, CA) | 1;100  1:1000 | 1:50  ND |
| DLK1 | Rabbit | Abcam (San Francisco, CA) | 1:1000 | 1:500 |
| FABP4 | Rabbit | Abcam (San Francisco, CA) | 1:1000 | ND |
| LPL  SIRT1  PGC-1  AKT1  pAKT1 | Mouse  Mouse  Rabbit  Rabbit  Rabbit | Cell Signaling Technology (Danvers, MA) Cell Signaling Technology (Danvers, MA)  Abcam (San Francisco, CA)  Abcam (San Francisco, CA)  Abcam (San Francisco, CA) | 1;100  1:1000  1:1000  1:2000  1:5000 | 1:50  ND  ND  ND  ND |

ND = Not detected; WB = Western blot; HS = Histochemical staining.
