# Supplementary figures and images for: Fibroblast growth factor homologous factor 1 stimulates Leydig cell regeneration from stem cells in male rats
Source: J Cell Mol Med. 2019 Jun 20;23(8):5618–31. doi: 10.1111/jcmm.14461 (PMC6653537; doi:10.1111/jcmm.14461)

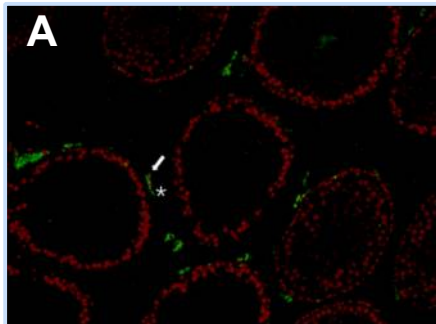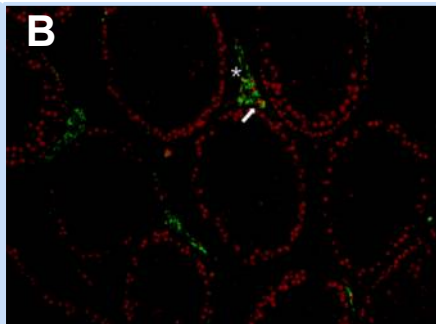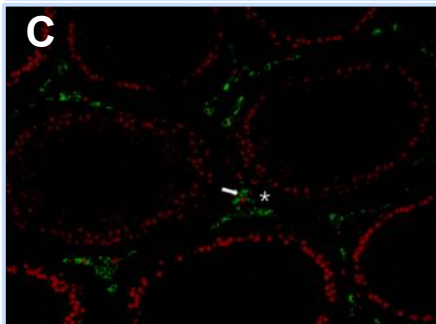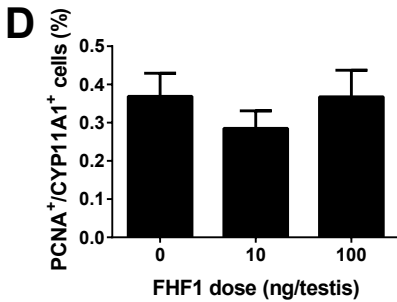

Supplement: Supplementary file 4 [file JCMM-23-5618-s004.pdf]

Count

0

10

100

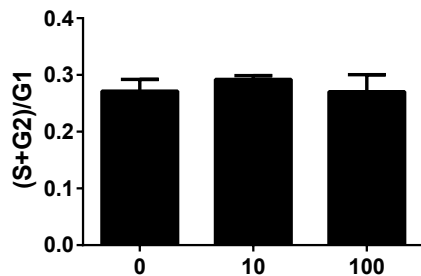

FHF1 (ng/ml)

Supplement: Supplementary file 5 [file JCMM-23-5618-s005.pdf]
